# Associations Between Dietary Carotenoid Intake and Plasma Carotenoid Concentrations and Skin Yellowness, a Validation Study

**DOI:** 10.1111/jhn.70075

**Published:** 2025-06-16

**Authors:** Erin D. Clarke, Mitch J. Duncan, Tracy Burrows, María Gómez‐Martín, Katherine Brain, Jordan Stanford, Jessica J. A. Ferguson, Lisa Wood, Clare E. Collins

**Affiliations:** ^1^ School of Health Sciences, College of Health, Medicine and Wellbeing University of Newcastle Callaghan New South Wales Australia; ^2^ Food and Nutrition Research Program Hunter Medical Research Institute New Lambton Heights New South Wales Australia; ^3^ School of Medicine and Public Health, College of Health, Medicine and Wellbeing University of Newcastle Callaghan New South Wales Australia; ^4^ School of Biomedical Sciences and Pharmacy University of Newcastle Callaghan New South Wales Australia

**Keywords:** dietary assessment, dietary carotenoids, skin carotenoids, validation study

## Abstract

**Background:**

Carotenoids are pigments abundant in fruits and vegetables and can be measured in plasma and skin. This study aimed to evaluate associations between carotenoid intake, assessed by self‐reported usual diet against plasma carotenoid and skin yellowness concentrations in Australian adults (*n* = 50, aged 39.3 ± 15.4 years, 68% female).

**Methods:**

Dietary carotenoid intakes were quantified using the Australian Eating Survey (AES) food frequency questionnaire (total, α‐ and β‐carotene, lycopene, β‐cryptoxanthin, and lutein/zeaxanthin). Plasma concentrations of these carotenoids were measured using high‐performance liquid chromatography, while skin yellowness was measured using skin reflectance spectroscopy as a proxy for skin carotenoids. Associations between AES carotenoids, plasma carotenoids and total skin yellowness were analysed using linear regressions.

**Results:**

AES‐derived carotenoid intakes were positively associated with skin yellowness for all dietary carotenoids (*β* range 0.25–0.46, *p* < 0.05) and total dietary carotenoids (*β* = 0.35 [95% CI 0.07, 0.63], *p* < 0.05), except lycopene. Similarly, each individual plasma carotenoid was significantly positively associated with their respective individual dietary carotenoids (*β* range 0.42–0.53, *p* < 0.05) and total dietary carotenoids (*β* = 0.38 [95% CI 0.04, 0.73], *p* < 0.05), except for lycopene. Significant positive associations were identified between skin yellowness and total (*β* = 0.36 [0.20, 0.52], *p* < 0.001) and individual plasma carotenoids (*β* range 0.30–0.37, *p* < 0.01), excluding α‐carotene and lycopene.

**Conclusion:**

Dietary carotenoid intakes were significantly associated with plasma carotenoid concentrations and skin yellowness values. Results support use of all three methods for assessment of carotenoid intake, with the exception of lycopene. Future studies should consider cost, accessibility of assessment methods and participant burden when selecting dietary assessment methods.

**Trial Registration:**

The original study was registered with Australian New Zealand Clinical Trials Registry (ANZCTR‐12619001415190).

## Summary

- Dietary intake of carotenoids, excluding lycopene, was significantly associated with plasma carotenoid concentrations and skin yellowness.
- The Australian Eating Survey food frequency questionnaire, plasma carotenoids and skin yellowness are all methods that can be used to assess carotenoid intake in Australian adults.
- When choosing a dietary assessment method for measuring carotenoid intakes consideration to cost, accessibility of assessment method and participant burden should be considered.

**Abbreviations**
AES: Australian Eating Survey
AUSNUT: AUStralian Food and NUTrient Database
FFQ: food frequency questionnaire
USDA‐NCI: United States Department of Agriculture and National Cancer Institute

## 1 Introduction

Poor quality diets, including inadequate intake of fruits and vegetables, contributed to 11 million deaths globally in 2017, with low fruit intake attributing to 2 million global deaths that same year [1]. It has been speculated that specific phytonutrients, such as carotenoids, found in fruits and vegetables, contribute to their beneficial effects, including antioxidant properties which reduce inflammation in association with reduced risk of chronic disease [2, 3]. Carotenoids are fat‐soluble pigments which confer fruits and vegetables with their red, yellow and orange colour [4]. There are several types of carotenoids found in varying amounts in different fruits and vegetables, with the most common being β‐carotene, α‐carotene, β‐cryptoxanthin, lycopene, lutein and zeaxanthin. For example, carrots, leafy vegetables, pumpkin and sweet potatoes are rich in β‐carotene, while tomatoes, pink grapefruit and watermelon are a good source of lycopene [5, 6]. After consumption of carotenoid‐rich foods, carotenoids are absorbed via the small intestine into the bloodstream where they are transported to target tissues, including the skin [7]. Circulating plasma and skin carotenoids have previously been identified as biomarkers of dietary carotenoid intake, fruit and vegetable intakes and a marker of overall diet quality [7, 8, 9, 10, 11].

Online food frequency questionnaires (FFQs) can be a useful method for dietary assessment as they are easy to administer, cost effective, do not require a trained interviewers and can be used in large cohorts [12, 13]. Compared to paper‐based FFQs, online administration can increase response rates without compromising data quality and can generate immediate analysis of food and nutrient intakes that can populate nutrition reports [14, 15, 16]. Prior validation studies of online FFQs have indicated validity for self‐reported nutrient, individual food and food group intakes when assessing dietary intake compared to traditional paper‐based FFQs [15, 16]. However, online FFQs still rely on self‐report and are subject to the same risk of bias and misreporting [13]. These issues can be addressed by using biomarker measurements to complement self‐reported dietary assessment techniques and improve accuracy of dietary assessment [8, 17]. Using biomarkers, such as skin yellowness and plasma carotenoids concentrations, in combination with traditional dietary assessment methods allows for a greater understanding of error when evaluating relationships between dietary intakes, health and impact of dietary interventions [18, 19].

Internationally, over a hundred studies have assessed the utility of plasma carotenoids as biomarkers of carotenoid intake [8], while fewer studies (*n* = 13) have validated technology‐based dietary assessment methods, such as online FFQs against skin carotenoids measured by reflectance spectroscopy or plasma carotenoid biomarkers [17]. Importantly, to our knowledge, only one other study has assessed dietary carotenoid intake quantified using an online FFQ, against both skin yellowness measured by reflectance spectroscopy and plasma carotenoid concentrations, which was conducted in young women only [7]. Previous research in Australian cohorts have identified significant relationships between skin yellowness, measured using skin reflectance spectroscopy and carotenoid intakes or brief diet quality scores; however, this has been previously assessed in young adults only and not using the online version of the FFQ [9, 11]. Further research to delineate the role and alignment of skin yellowness assessment via skin reflectance spectroscopy as an objective measure of carotenoid intake is needed. While feasibility may depend on the availability of appropriate equipment, it poses as an attractive method that is easily implemented, noninvasive, provides immediate results with lower burden on researchers/participants compared to plasma carotenoid levels, and potentially comparable or increased accuracy compared to diet‐derived carotenoid intake data.

Therefore, the aim of the current study was to determine the relationships between plasma carotenoids concentrations and skin yellowness, as an indicator of skin carotenoids measured by reflectance spectroscopy, as biomarkers of self‐reported dietary carotenoid intakes quantified from an online FFQ in a group of Australian adults aged 18 years and older.

## 2 Materials and Methods

### 2.1 Participants and Recruitment

The current study reports cross‐sectional findings of data from participants recruited in the Newcastle region, NSW, between September 2019 and March 2020. Participants completed the Australian Eating Survey FFQ, anthropometric measures, demographic questionnaires, skin reflectance spectroscopy assessment and blood sample collection. Eligible participants were 18 years or older, weight stable (±4 kg) in the past 2 months, had access to the internet and were able to travel to The University of Newcastle to attend data collection sessions. This study was conducted according to the guidelines laid down in the Declaration of Helsinki and all procedures involving human subjects were approved by The University of Newcastle Human Research Ethics Committee (H‐2019‐0147) and the original study was registered Australian New Zealand Clinical Trials Registry (ANZCTR‐12619001415190). All participants provided written informed consent. Reporting was guided by the STROBE‐NUT guidelines (Supporting Information S1: S1) [20]. Sample size was determined by the feasibility of recruitment due to constraints of COVID‐19.

### 2.2 Dietary Assessment Methods

Dietary intake was assessed using the Australian Eating Survey (AES), a 120‐item FFQ, administered online to capture usual intake over the last 3 months [21]. The AES also captures some demographic characteristics with the entire questionnaire taking ~20–30 min to complete. Previously, validation studies using the AES have been conducted to evaluate the relationship between fruit, vegetables and dietary carotenoid intakes with plasma carotenoid concentrations and skin yellowness measures, as a proxy for skin carotenoid content in young women only [7]. However, the current study will quantify this relationship in a sample of older male and female Australian adults.

Nutrient intakes were calculated using the AUSNUT 2011‐2013 database, which provides estimates for β‐carotene but lacks data on other carotenoids [22]. To obtain data on other carotenoids, the US Department of Agriculture and the National Cancer Institute (USDA‐NCI) [23], using methods described previously [24], was also applied to the AES data. In summary, β‐carotene intakes were quantified from both the AUSNUT and USDA‐NCI databases, denoted as ‘β‐carotene (AUSNUT)’ and ‘β‐carotene (USDA‐NCI)’, respectively throughout the manuscript. However, α‐carotene, lycopene, cryptoxanthin, combined lutein/zeaxanthin and total carotenoid intakes were estimated using only the USDA‐NCI database [23].

Fruit and vegetable intakes were calculated based on 11 questions relating to fruit and 21 questions relating to vegetable intake. Serving sizes were calculated by summing total weight of fruit and vegetables consumed using the AES divided by a standard serving size as defined in the Australian Guide to Healthy Eating (fruit serve = 150 g, vegetable serve = 75 g) [25]. While information on supplement usage was collected, these data were not included in these analyses.

### 2.3 Blood Collection and Plasma Carotenoid Analysis

Participants attended clinical assessment sessions after an overnight fast. Blood samples were collected in EDTA‐coated tubes by a trained phlebotomist at an accredited pathology service. Samples were processed by the pathology service before being stored at −80°C until analysis.

High‐performance liquid chromatography was undertaken to determine plasma carotenoid concentrations of β‐carotene, lycopene, β‐cryptoxanthin, α‐carotene and lutein/zeaxanthin, as described previously [26, 27]. A darkened laboratory under red light was used to carry out all extractions. Carotenoids were isolated from plasma by adding ethanol:ethyl acetate (1:1) containing internal standards (canthaxanthin) and butylated hydroxytoluene before being vortexed and centrifuged at 3000*g* at 48°C for 5 min, and the supernatant collected. Three more times this process was repeated, with ethyl acetate being added twice and then hexane to the pellet. Ultrapure water was added to the supernatant before the mixture was vortexed and centrifuged. Again, the supernatant was decanted and placed on a nitrogen evaporator until solvents completely evaporated. The dried extract was reconstituted in dichloromethane:methanol (1:2). Chromatography was performed using an Agilent 1200 series liquid chromatograph (Agilent Technologies, Santa Clara, CA, USA, part No. G1311‐90011) using a Hypersil ODS column (100 mm × 2.1 mm × 5 µm). Chemstation data analysis software at a flow rate of 0.3 mL/min was used (Chemstation OpenLab CDS software, Agilent Technologies, Melbourne, VIC, Australia). Analysis used the mobile phase of acetonitrile:dichloromethane:methanol 0.05% ammonium acetate (85:10:5 vol/vol) and a diode array detector (450 nm). β‐Carotene, lycopene, β‐cryptoxanthin, α‐carotene and lutein/zeaxanthin were summed to calculate total plasma carotenoids.

### 2.4 Skin Reflectance Spectroscopy Analysis of Skin Yellowness

Skin yellowness as a marker of skin carotenoids was measured using skin reflectance spectroscopy (CM700D, Konica Minolta). Previous studies in young women and young adults have validated the use of skin reflectance spectroscopy as a noninvasive method to assess dietary carotenoids, fruit and vegetable intakes [9, 11], these methods have been replicated in the current study. Briefly, before each assessment, a white calibration was undertaken using the spectral reflectance of a white calibration tile that is included as part of the CM700D model. Measures were then taken from the sanitised palm of the left hand, the left inner and outer arm. Each site was measured three times at each clinic visit and the average calculated. For each measure, L* (lightness), a* (redness) and b* (yellowness) values were recorded. The primary value used was skin yellowness (b* values), previously shown to be significantly associated with dietary carotenoid intakes [9, 11].

### 2.5 Statistical Analysis

The AES‐derived carotenoids were classified into tertiles and weighted Kappa statistics were used to assess the level of agreement between the AES derived carotenoid, fruit and/or vegetable intakes and total skin and plasma carotenoids. Because FFQs are typically used to rank participants categorically into high versus low consumers, weighted Kappa statistics were chosen. Additionally, Kappa statistics take into account the agreement that could occur by chance when looking only at associations [28]. The magnitude of agreement was classified as Kappa coefficient: ≤ 0 = poor, 0.01–0.20 = slight, 0.21–0.40 = fair, 0.41–0.60 = moderate, 0.61–0.80 = substantial, and 0.81–1 = almost perfect [29].

As the AES‐derived carotenoids, skin yellowness and plasma carotenoids continuous measures use different metrics (i.e., μg/day for AES, b* for the skin colouration, μg/dL for the plasma carotenoids), the agreement and bias between the three measures were assessed using plots of AES‐derived carotenoid versus skin yellowness and AES‐derived carotenoid versus plasma carotenoids with 95% prediction intervals fitted [30].

The relationship between AES‐derived carotenoids, total skin yellowness and plasma carotenoids was assessed using linear regression. For each AES derived carotenoid, unadjusted and adjusted regression models were examined. Adjusted models included potential confounders identified from previous research [11], and included age, gender, skin lightness [11], total energy intake, fat intake and BMI [11]. The selection of the confounders adjusted for in analyses was identified based on being associated with either the exposure and/or outcome using a conservative *p* value of 0.250 and the magnitude of the association, and also a change in estimates approach [31]. To facilitate comparison between the carotenoids using different metrics, before analysis the variables were standardised ([value – mean]/standard deviation) to obtain standardised regression coefficients, standard errors and confidence intervals. Residual diagnostics were used to check assumptions and identify potential outliers. As some heteroskedasticity was observed regression analyses used robust standard errors, with no other noticeable violations observed. Exclusion of outliers from analyses did not appreciably change the interpretation of the results and so were retained for completeness. Statistical analyses were conducted using Stata MP 17 (StataCorp. Stata Statistical Software: Release 17. College Station, TX: Stata Corp LLC).

## 3 Results

### 3.1 Sample Characteristics

The mean (SD) participant age was 39.6 ± 15.5 years, the average BMI was 25.7 ± 5.4 kg/m^2^ (range = 27.7) and was predominantly female (68.0%), Caucasian (86.0%) and reported at least a university education (50.0%) (Table 1). Of the 59 participants, 50 had complete data for dietary intake, skin reflectance and plasma carotenoid values.

**Table 1:** Participant demographic, dietary and plasma carotenoid and skin yellowness characteristics (*n* = 50).

	Total (*n* = 50)	
	Mean, *N*	(SD) %
Socio‐demographic and behavioural		
Age (years)	39.6	(15.5)
Gender		
Male	16	32.0%
Female	34	68.0%
Ethnicity categories		
Caucasian	43	86.0%
Asian	4	8.0%
Mixed/Other	2	4.0%
Missing	1	2.0%
Indigenous status		
No	47	94.0%
Aboriginal	2	4.0%
Missing	1	2.0%
Education categories		
High school/No formal education	15	30.0%
Trade	10	20.0%
University	25	50.0%
Employment categories		
Full time (≥ 35 h/week)	18	36.0%
Part time (< 35 h/week)/Casual	18	36.0%
Student, not employed, prefer not to say	14	28.0%
BMI (kg/m^2^)	25.7	(5.4)
Height (cm)	169.9	(8.4)
Weight (kg)	74.4	(17.9)
Smoking status		
No	47	94.0%
Yes	3	6.0%
Comorbidities/medical conditions		
None	31	62.0%
1 reported	13	26.0%
2+ reported	6	12.0%
AES dietary intake		
α‐Carotene (μg/day)	2050	(1360)
β‐Carotene (AUSNUT) (μg/day)	7469	(3948)
β‐Carotene (USDA‐NCI) (μg/day)	3921	(2176)
β‐Cryptoxanthin (μg/day)	573	(450)
Lutein/zeaxanthin (μg/day)	2883	(1666)
Lycopene (μg/day)	4398	(2438)
Fruit (g/day)	279	(204)
Vegμetables (g/day)	369	(174)
Plasma		
Lutein/Zeaxanthin (μg/dL)	0.4	(0.2)
β‐Cryptoxanthin (μg/dL)	0.3	(0.2)
Lycopene (μg/dL)	0.4	(0.3)
α‐Carotene (μg/dL)	0.1	(0.1)
β‐Carotene (μg/dL)	0.4	(0.5)
Total plasma carotenoids (μg/dL)	1.5	(1.1)
Skin coloration reflectance spectroscopy		
Total lightness (L*)	63.4	(5.6)
Total redness (a*)	9.8	(3.1)
Total yellowness (b*)	17.9	(2.4)

Abbreviations: AUSNUT, AUStralian Food and NUTrient Database; USDA‐NCI, United States Department of Agriculture and National Cancer Institute.

### 3.2 The Level of Agreement Between Self‐Reported Dietary Carotenoids, Fruits and Vegetables, Skin Yellowness and Plasma Carotenoids

Level of agreement between AES derived dietary carotenoids and skin yellowness measures indicated fair agreement between the measures for α‐carotene, β‐carotene, and lutein/zeaxanthin (Table 2). The agreement between β‐cryptoxanthin and skin yellowness was slight and there was poor agreement between lycopene and skin yellowness (Table 2). Agreement between AES‐derived carotenoids and the respective plasma carotenoids measures is presented in Table 2, with all AES‐derived carotenoids showing fair agreement, except for lycopene. Agreement between AES‐derived carotenoids and total plasma carotenoids indicated slight agreement for each individual AES dietary carotenoid with total plasma carotenoids, and fair agreement between total AES dietary carotenoids and total plasma carotenoids (Table 2).

**Table 2:** Agreement between AES carotenoids with total skin yellowness and plasma carotenoids.

	Skin yellowness			Specific plasma carotenoid			Total plasma carotenoids		
	Expected %	Observed %	κ	Expected %	Observed %	κ	Expected %	Observed %	κ
α‐Carotene	0.55	0.66	0.24	0.55	0.71	0.35	0.55	0.63	0.17
β‐Carotene (AUSNUT)	0.57	0.72	0.35	0.57	0.70	0.30	0.58	0.65	0.17
β‐Carotene (USDA‐NCI)	0.57	0.71	0.33	0.57	0.71	0.32	0.58	0.66	0.19
β‐Cryptoxanthin	0.55	0.58	0.06	0.55	0.65	0.23	0.57	0.59	0.05
Lutein/Zeaxanthin	0.56	0.65	0.21	0.55	0.68	0.28	0.56	0.60	0.09
Lycopene	0.56	0.50	−0.15	0.57	0.58	0.03	0.60	0.59	−0.03
Total AES carotenoids	0.58	0.72	0.33				0.68	0.75	0.22

*Note:* Number of observations is 50. Agreement is denoted by Kappa coefficients: ≤ 0 = poor, 0.01–0.20 = slight, 0.21–0.40 = fair, 0.41–0.60 = moderate, 0.61–0.80 = substantial, and .81–1 = almost perfect [29]. Agreement between the specific AES carotenoid and total skin yellowness is reported in the Skin Yellowness column. Agreement between the specific AES carotenoid and the respective measured plasma carotenoid is reported in the Specific Plasma Carotenoid column (e.g. AES α‐Carotene with Plasma α‐Carotene). Agreement between the specific AES carotenoid and the total measured plasma carotenoid (calculated as the sum of plasma carotenoids) is reported in the Total Plasma Carotenoid column (e.g. AES α‐Carotene with Total Plasma carotenoids).

Abbreviations: AUSNUT, AUStralian Food and NUTrient Database; USDA‐NCI, United States Department of Agriculture and National Cancer Institute.

**Figure 1:**
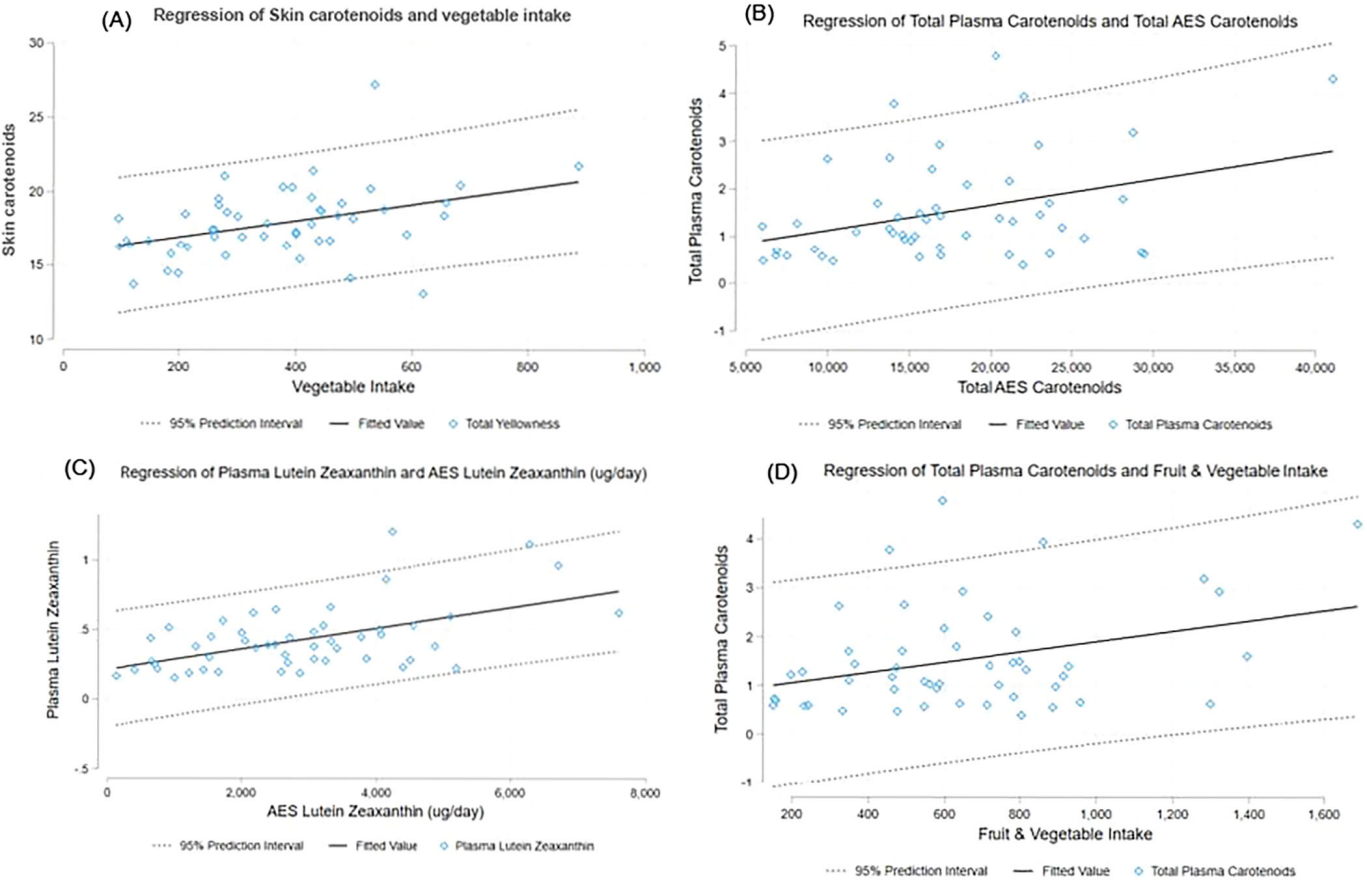
Regression Models. (A) Total skin yellowness and vegetable intake. (B) Total plasma carotenoids and total AES carotenoids. (C) Plasma lutein/zeaxanthin and AES lutein/zeaxanthin. (D) Total plasma carotenoids and fruit + vegetable intakes.

The level of agreement (Kappa coefficients) between fruit intakes, skin yellowness, and plasma carotenoids was primarily slight to fair (Table 3). The greatest level of agreement was observed for fruit intake and plasma lutein/zeaxanthin. The level of agreement for vegetable intake was mostly higher, with the majority classified as fair to moderate (Table 3 and Figure 1). The strongest level of agreement was between total vegetable intake and skin yellowness. As expected, the level of agreement for combined fruit and vegetable intakes was between that of fruits and vegetables alone (Table 3). Again, the greatest level of agreement observed was for combined fruit and vegetables intakes and skin yellowness.

**Table 3:** Agreement between AES fruit and vegetable intake with total skin yellowness, plasma carotenoids, and AES carotenoids.

	Fruit intake			Vegetable intake			Fruit and vegetable intake		
	Expected %	Observed %	*κ*	Expected %	Observed %	*κ*	Expected %	Observed %	*κ*
Skin yellowness	0.56	0.61	0.11	0.55	0.71	0.36	0.56	0.67	0.26
Plasma carotenoids									
α‐Carotene	0.56	0.60	0.08	0.55	0.64	0.20	0.55	0.58	0.06
β‐Carotene	0.57	0.59	0.06	0.55	0.63	0.18	0.56	0.59	0.07
β‐Cryptoxanthin	0.55	0.60	0.10	0.54	0.52	−0.05	0.55	0.60	0.11
Lutein/Zeaxanthin	0.56	0.68	0.27	0.55	0.60	0.12	0.55	0.64	0.19
Lycopene	0.56	0.53	−0.07	0.55	0.57	0.05	0.55	0.55	−0.01
Total carotenoids	0.55	0.58	0.06	0.55	0.56	0.02	0.56	0.62	0.13

*Note:* Number of observations is 50. Agreement is denoted by Kappa coefficients: ≤ 0 = poor, 0.01–0.20 = slight, 0.21–0.40 = fair, 0.41–0.60 = moderate, 0.61–0.80 = substantial, and 0.81–1 = almost perfect [29]. Agreement between the AES fruit intake (grams), vegetable intake (grams), and total AES fruit and vegetable intake (grams) with total skin yellowness and the respective measured plasma carotenoid.

### 3.3 The Relationship Between Dietary Carotenoids and Skin Yellowness

Dietary intakes of AES‐derived carotenoids were positively associated with total skin yellowness for all measures (noting adjusted association for β‐cryptoxanthin was just borderline, *p* = 0.047), except lycopene, which demonstrated no significant association with skin yellowness. The unadjusted and adjusted associations between AES‐derived carotenoids and total skin yellowness were of small to moderate magnitude (Table 4). Significant positive associations remained between each individual and total dietary carotenoid and skin yellowness in the adjusted models, except for lycopene and lutein/zeaxanthin.

**Table 4:** Unadjusted and adjusted associations between AES‐derived carotenoids and total skin yellowness (b* values).

	Unadjusted				Adjusted			
	*B*	SE	(95% CI)	*p*	B	SE	(95% CI)	*p*
α‐Carotene	0.26	0.14	(−0.02, 0.53)	0.07	**0.46**	**0.09**	**(0.28, 0.63)**	**< 0.001**
β‐Carotene (AUSNUT)	**0.34**	**0.10**	**(0.13, 0.55)**	**0.002**	**0.46**	**0.10**	**(0.25, 0.67)**	**< 0.001**
β‐Carotene(USDA‐NCI)	**0.28**	**0.13**	**(0.02, 0.55)**	**0.04**	**0.41**	**0.12**	**(0.16, 0.66)**	**0.002**
β‐Cryptoxanthin	0.15	0.14	(−0.14, 0.44)	0.30	**0.25**	**0.12**	**(0.00, 0.50)**	**0.05**
Lutein/zeaxanthin	**0.33**	**0.14**	**(0.04, 0.62)**	**0.03**	0.26	0.16	(−0.06, 0.58)	0.11
Lycopene	−0.25	0.16	(−0.58, 0.08)	0.14	−0.20	0.19	(−0.59, 0.18)	0.30
Total dietary carotenoids	0.21	0.15	(−0.10, 0.51)	0.18	**0.35**	**0.14**	**(0.07, 0.63)**	**0.02**

*Note:* Variables standardised and centred before analysis. *B* are standardised regression coefficients. *B* of < 0.2 were classified as weak, 0.2–0.6 moderate and > 0.6 strong based on previous studies [11]. Adjusted analyses adjusted for skin lightness (L* value) and fat intake (g/day). Robust standard errors applied.

Abbreviations: AUSNUT, AUStralian Food and NUTrient Database; USDA‐NCI, United States Department of Agriculture and National Cancer Institute.

### 3.4 The Relationship Between Dietary Carotenoids and Plasma Carotenoids

All AES‐derived carotenoids had a statistically significant positive relationship with their respective plasma carotenoids, except lycopene, which demonstrated no significant association. Again, all AES‐derived carotenoids showed a positive relationship with total plasma carotenoids (Figure 1), except for lycopene. The unadjusted and adjusted associations between AES‐derived carotenoids and plasma carotenoids were moderate in magnitude (Table 5) and the 95% confidence intervals indicate the variability in the AES‐derived carotenoids. Overall, both unadjusted and adjusted models for each individual and total dietary carotenoid with their respective plasma carotenoid were significantly, positively associated, with the exception of lycopene.

**Table 5:** Unadjusted and adjusted associations between AES‐derived carotenoids and plasma carotenoids.

	Unadjusted				Adjusted			
	*B*	SE	(95% CI)	*p*	*B*	SE	(95% CI)	*p*
α‐Carotene	**0.47**	**0.08**	**(0.30, 0.64)**	**< 0.001**	**0.45**	**0.10**	**(0.25, 0.64)**	**< 0.001**
β‐Carotene (AUSNUT)	**0.49**	**0.11**	**(0.26, 0.72)**	**< 0.001**	**0.50**	**0.12**	**(0.25, 0.75)**	**< 0.001**
β‐Carotene (USDA‐NCI)	**0.43**	**0.14**	**(0.15, 0.70)**	**0.003**	**0.42**	**0.13**	**(0.16, 0.69)**	**0.003**
β‐Cryptoxanthin	**0.51**	**0.18**	**(0.15, 0.87)**	**0.007**	**0.45**	**0.20**	**(0.04, 0.86)**	**0.03**
Lutein/zeaxanthin	**0.54**	**0.15**	**(0.23, 0.84)**	**< 0.001**	**0.53**	**0.15**	**(0.22, 0.83)**	**0.001**
Lycopene	−0.03	0.15	(−0.33, 0.26)	0.82	0.05	0.15	(−0.25, 0.36)	0.72
Total carotenoids	**0.36**	**0.17**	**(0.02, 0.70)**	**0.04**	**0.38**	**0.17**	**(0.04, 0.73)**	**0.03**

Abbreviations: AUSNUT, AUStralian Food and NUTrient Database; USDA‐NCI, United States Department of Agriculture and National Cancer Institute.

*Note:* Variables standardised and centred before analysis. *B* are standardised regression coefficients. *B* of < 0.2 were classified as weak, 0.2–0.6 moderate and > 0.6 strong based on previous studies [11]. Adjusted analyses adjusted for total energy intake (kJ/day), fat intake (g/day) and BMI (kg/m^2^). Robust standard errors applied. Associations are reported between the specific AES‐derived carotenoid and the respective measured plasma carotenoid.

### 3.5 The Relationship Between Skin Yellowness and Plasma Carotenoids

The unadjusted and adjusted associations between skin yellowness and plasma carotenoids were moderate in magnitude (Table 6). Significant positive associations were identified between skin yellowness and both total and specific carotenoids, with the exception of α‐carotene and lycopene.

**Table 6:** Unadjusted and adjusted associations between plasma carotenoid concentrations and skin yellowness (b* value).

	Unadjusted				Adjusted			
	*B*	SE	(95% CI)	*p*	*B*	SE	(95% CI)	*p*
α‐Carotene	0.31	0.31	(−0.31, 0.93)	0.32	0.28	0.25	(−0.22, 0.78)	0.26
β‐Carotene	**0.42**	**0.16**	**(0.09, 0.74)**	**0.01**	**0.37**	**0.12**	**(0.13, 0.60)**	**0.003**
β‐Cryptoxanthin	**0.36**	**0.13**	**(0.09, 0.63)**	**0.010**	**0.30**	**0.10**	**(0.09, 0.51)**	**0.006**
Lutein	**0.43**	**0.09**	**(0.26, 0.61)**	**< 0.001**	**0.32**	**0.09**	**(0.13, 0.50)**	**0.002**
Lycopene	0.17	0.13	(−0.10, 0.44)	0.20	0.16	0.08	(−0.00, 0.32)	0.06
Total carotenoids	**0.41**	**0.12**	**(0.17, 0.65)**	**0.001**	**0.36**	**0.08**	**(0.20, 0.52)**	**< 0.001**

*Note:* Variables standardised and centred before analysis. *B* are standardised regression coefficients. *B* of < 0.2 were classified as weak, 0.2–0.6 moderate and > 0.6 strong based on previous studies [11]. Adjusted analyses adjusted for fat intake (g/day), skin lightness (L* value) and BMI (kg/m^2^). Robust standard errors applied. Bold values indicate statistically significant.

## 4 Discussion

The current study identified in a wider age range sample of Australian adults that dietary carotenoid intakes, except for lycopene, were significantly related to skin yellowness values and plasma carotenoid concentrations. These findings indicate that the AES FFQ completed online can be used to quantify longer term (3 months) intakes of dietary carotenoids in Australian adults, with both skin yellowness (measured via skin reflectance spectroscopy) and plasma carotenoid concentrations being able to be used as objective measures of longer‐term dietary intake of fruits and vegetables in Australian adults, with the exception of lycopene. These findings contribute towards the existing evidence available for young Australian adults and young Australian women for which data for Australian males and/or older Australians are scarce.

The overall level of agreement between dietary‐derived carotenoid intake and skin yellowness was slight to fair for all carotenoids except for lycopene. To our knowledge, few studies have examined the level of agreement between dietary intake and skin yellowness. Ashton et al. (2017) reported similar Kappa agreement relationships in young (18–25 years) Australian adults (*K* 0.14–0.24) between intake of fruit and vegetable variety (also measured using the AES FFQ) and skin yellowness [11]. While the exact relationship explored (variety vs*.* total intake) was different as that examined in the current study, fruits and vegetables are rich in carotenoids and contribute to dietary carotenoid intakes [4, 5], which may explain why similar level of agreement was observed between studies especially when the same dietary assessment tool was used. Additionally, prior research using the AES identified that there was a poor level of agreement between fruit and vegetable variety and lycopene intake, but slight to fair relationships for all other carotenoids [32]. An explanation for these results could be the large interindividual variability in lycopene intakes, influenced by several factors such as food preparation procedures, differential bioavailability depending on co‐consumption of dietary fat, soluble fibre, preferential oxidation of lycopene in the skin, lycopene a red carotenoid having a higher absorption spectrum (400–750 nm spectral rage [33], the higher end of the Konica Minolta spectrophotometer range [11]) than yellow‐orange carotenoids (400–540 nm) [9], and/or differences in genetic polymorphisms associated with lycopene absorption and metabolism [34, 35, 36, 37]. Interestingly, some authors found minimal increases in plasma lycopene after consumption of lycopene‐rich food when consumed without fat [38, 39, 40]. A recent review suggests that optimal lycopene absorption requires a minimum of 10 g of fat in a meal containing processed tomatoes, while 15 g may be needed for uncooked foods such as salads or raw tomatoes [41]. This indicates that lycopene levels depend not only on the consumption of lycopene‐rich foods but also a minimum threshold of fat, which facilitates lycopene bioavailability absorption across various food matrices [41]. Furthermore, several single‐nucleotide polymorphisms have been identified in genes that code for proteins involved in carotenoid intestinal uptake, transport and metabolism [42, 43]. While genetic polymorphisms and bioavailability status, including optimal dietary fat thresholds, were outside the scope of the current study, they should be acknowledged as potential factors influencing the lack of associations observed between objective carotenoid intake and lycopene.

The level of agreement between fruit and/or vegetable intakes and plasma and skin yellowness varied. Generally, stronger Kappa coefficients were identified for vegetable intakes than fruit intakes and carotenoid biomarkers. This may be due to the nature of consumption of vegetables in the diet, which often involves cooking with oils/fats which increases carotenoid bioavailability [44] and bio‐accessibility [45]. Skin yellowness was identified as the strongest biomarkers for vegetable and combined fruit and vegetable intakes. This finding aligns with prior research, which has shown that skin yellowness measured by skin reflectance spectroscopy is a valid biomarker of fruit and vegetable intakes [7, 9, 17]. On the other hand, plasma lutein/zeaxanthin had the strongest relationship with fruit intakes, again this aligns with prior research which has shown that plasma lutein/zeaxanthin is associated with fruit intake [8, 46, 47, 48]. Interestingly, we continue to see poor or nonsignificant relationships between fruit and vegetable intakes and plasma lycopene which is consistent with prior work [7, 24] and is likely due to lycopene being consumed in large quantities in non‐fruit and vegetable sources, for example, pizza, ketchup. These findings support the use of skin reflectance spectroscopy assessment as a quantification of skin yellowness as well as for validation of vegetables or combined fruit and vegetable intakes. Plasma lutein/zeaxanthin may be used as a biomarker of fruit intake; however, it is important to consider the types of fruit eaten in different populations as this may vary the strength of relationships identified.

Dietary carotenoid intakes, excluding lycopene, were significantly associated with skin yellowness, with every 0.35 μg/day intake in total dietary carotenoids resulting in a 1‐point increase in skin yellowness outcome. The relationships identified in the current study are not similar to those reported in other studies conducted in Australia using the same methodology [9]. The study by Pezdirc et al. was conducted in young adult females and found that only dietary intake of lycopene and lutein/zeaxanthin were significantly associated with skin yellowness outcome [9], which contrasts what was observed in the current study using a wider age range of adults and including both males and females with dietary intakes of lycopene and lutein/zeaxanthin having no association with skin yellowness outcome. Results from the current study may differ due to population differences in age, sex and BMI. Carotenoids are known to play a role in reducing the rate of aging and circulating levels of carotenoids are lower at a higher body fat mass due to being sequestered in adipose tissue [49, 50, 51], but the molecular mechanisms of how and why this occurs are not fully elucidated [49]. Results from a systematic review found that studies measuring skin yellowness using spectrophotometers, such as the CM700D, have shown significant relationships between dietary carotenoid intakes and skin yellowness values, with weaker correlations identified between specific individual carotenoids, including lycopene [52]. Of note, this review identified that skin yellowness has primarily been assessed using resonance Raman spectroscopy (RRS) technology and few have used pressure mediated reflectance spectroscopy using the Veggie Meter device [52]. Compared to studies included in this review that used different spectroscopy devices to assess skin carotenoids associations between RRS and total carotenoid intakes ranged from fair to moderate [53, 54, 55], similar to results reported in the current study. Stronger associations were found in the current study when comparing the relationship between individual carotenoids and skin yellowness than those found using RRS or pressure mediated reflectance devices, with the exception of lycopene and β‐cryptoxanthin [37, 55]. No studies in the review had assessed the relationship between skin carotenoids and self‐reported carotenoid intakes or plasma carotenoids measured using the Veggie Meter [52]. Findings from the current study add to the literature to support the use of skin yellowness assessment, measured using skin reflectance spectroscopy, as an indicator of dietary carotenoid intake.

Similar to skin yellowness results, significant associations were identified between plasma carotenoids and dietary carotenoids intakes. This is consistent with results from systematic reviews which reported skin yellowness as biomarkers of fruits and vegetables [52] and carotenoid intakes [17]. Both reviews reported significant relationships between derived carotenoid intakes and plasma carotenoid concentrations [17, 52]. Findings from the current study support the validity of the AES FFQ in quantifying dietary carotenoid intakes which had been previously validated against plasma carotenoids [24].

The associations identified between both objective measures of carotenoid intake, that is, skin yellowness and plasma carotenoids were similar to those observed between each objective measure and dietary carotenoid intakes. These results are promising as carotenoids cannot be produced endogenously and therefore must be supplied by the diet [52], highlighting that each method used is representative of actual dietary carotenoid consumption. While it is acknowledged that there are limitations to self‐reported dietary intakes, knowing that dietary assessment tools such as validated FFQs can validly quantify intake using biomarkers, such as skin and plasma carotenoids, is important for our understanding measurement error when evaluating relationships between dietary intake and health. Collecting plasma samples is invasive, burdensome and expensive, while skin reflectance spectroscopy may not be easily accessible to researchers. FFQs in comparison are a cost effective, accessible method that can be applied easily to a range of contexts [14]. Overall, each method has its place in furthering our understanding of the consumption of dietary carotenoids and their relationship with health.

Recommendations for future research and practice include specific investigation into the methods required for objectively quantifying lycopene in plasma carotenoids and/or skin yellowness. Such studies may require controlled dietary interventions with specified dietary fat thresholds, consideration of fat and soluble fibre co‐consumption, food preparation techniques, and genetic variation. Nonetheless, all dietary carotenoid assessment methods explored in the current study (skin, plasma and derived intake) could be used to assess carotenoid intakes, with the exception of lycopene. Similarly, the level of agreement for plasma carotenoids and skin yellowness and fruit and/or vegetable intakes was similar, with skin yellowness potentially a stronger biomarker of vegetable intakes than plasma. Therefore, the method recommended would be dependent on cost, accessibility of collection tools and participant burden. Both the AES FFQ and skin reflectance are less burdensome and invasive compared with plasma carotenoids; however, where feasible, using multiple assessment methods are recommended to ensure that these tools and biomarkers continue to be reflective of dietary intakes, particularly when considering the changes in the food environment over time.

The current study has several strengths and limitations. The primary strength of this study is the novelty of using multiple biomarkers of dietary carotenoid intake to validate dietary intake of carotenoids from an online AES FFQ from both the AUSNUT and USDA‐NCI databases. To our knowledge, no other studies have used three assessment measures in older adult population samples. These findings also build on the validity of skin yellowness measured using skin reflectance spectroscopy as an objective measure of dietary vegetable, fruit and carotenoid intakes in a broader Australian population, not just young adults where the existing literature is concentrated. Additional strengths include the novelty of this study being conducted in an Australian population who may eat different varieties of vegetables and fruit and hence different patterns of carotenoid intakes, compared to other populations where carotenoid biomarkers have been validated [56, 57]. For example, compared to Europe, which report average β‐carotene intakes of ~2900 μg/day (range of 1901–3907 μg/day) intakes in the current study suggest a greater intake of β‐carotene‐rich fruits and vegetables [56]. Future studies should consider reporting more detail on the foods that contribute to carotenoid intakes, such as that done by Coyle et al. [58] to further understand the differences between countries. Plasma carotenoid concentrations vary slightly compared to previously published results; however, lycopene, β‐carotene and lutein/zeaxanthin remain the top three contributors to total carotenoid values as reported in a meta‐analysis by Burrows et al. [57] Lastly, that skin yellowness was assessed using the Konica Minolta CM700D and reported similar results to studies using RRS or pressure mediated reflectance spectroscopy devices such as the Veggie Meter [37, 52]. Limitations of the current study include limited sample size due to the effects of COVID‐19 which affected recruitment. The AES FFQ does not specify food preparation or cooking methods when asking questions about food intake. While food preparation and certain cooking techniques can positively and negatively impact content of various carotenoids, this could not be controlled for in the current study nor delineated. Skin reflectance spectroscopy may not always be feasible if the equipment and training of personnel is not possible. Lastly, associations between skin yellowness and dietary carotenoid intake were stronger compared to other studies, suggesting that these results may not be representative of other population groups, with the sample in the current study being primarily Caucasian with usual vegetable intake close on average to meeting Australian Guide to Healthy Eating recommendations [25]. Therefore, future observational and intervention studies in a wider range of ethnicities are recommended particularly when examining skin yellowness as an objective measure of carotenoid intakes in non‐Caucasian populations.

## 5 Conclusion

Findings from the current study identified that dietary carotenoid intakes, derived from self‐reported usual dietary intake using an online FFQ were significantly associated with carotenoid biomarkers, including skin yellowness and plasma carotenoids concentrations, in a group of Australian adults. These findings indicate that all three methods can be used as a measure of longer‐term (3 months) carotenoid intakes, with the exception of lycopene, in Australian adults aged 18–70 years. Future studies should consider factors such as cost, accessibility of collection tools and participant burden when designing studies.

## Author Contributions

E.D.C., T.B., K.B., and C.E.C. conceived the study. E.D.C. was involved in drafting the manuscript. M.J.D. and L.W. were involved in conducting the analysis. K.B. was involved in collecting the data. All authors reviewed and approved the final version of the manuscript.

## Ethics Statement

The University of Newcastle Human Research Ethics Committee (H‐2019‐0147).

## Conflicts of Interest

Jessica Ferguson also holds separate part‐time employment at the Sanitarium Health Food Company who had no input into the study and are not financially supporting or sponsoring any part of this study.

## Peer Review

The peer review history for this article is available at https://www.webofscience.com/api/gateway/wos/peer-review/10.1111/jhn.70075.

## Supporting information

Supplement 1 1.

## Data Availability

The authors have nothing to report.
